# Sinus lifting before Le Fort I maxillary osteotomy: a suitable method for oral rehabilitation of edentulous patients with skelettal class-III conditions: review of the literature and report of a case

**DOI:** 10.1186/1746-160X-3-2

**Published:** 2007-01-04

**Authors:** Rita A Depprich, Jörg GK Handschel, Christian Naujoks, Tobias Hahn, Ulrich Meyer, Norbert R Kübler

**Affiliations:** 1 Department for Cranio- and Maxillofacial Surgery, Heinrich-Heine-University Düsseldorf, Moorenstr. 5, 40225 Düsseldorf, Germany

## Abstract

**Background:**

Functional rehabilitation of patients afflicted with severe mandibular and maxillary alveolar atrophy might be challenging especially in malformed patients.

**Methods:**

Treatment planning using sinus lifting and implant placement before Le Fort I maxillary osteotomy in a patient with severe mandibular and posterior maxillary alveolar atrophy and skelettal class-III conditions due to cleft palate are described.

**Results:**

A full functional and esthetic rehabilitation of the patient was achieved by a stepwise surgical approach performed through sinus lifting as the primary approach followed by implant placement and subsequent Le Fort I maxillary osteotomy to correct the maxillo-mandibular relation.

**Conclusion:**

Stabilisation of the maxillary complex by a sinus lifting procedure in combination with computer aided implant placement as preorthodontic planning procedure before Le Fort I maxillary osteotomy seems to be suitable in order to allow ideal oral rehabilitation especially in malformed patients.

## Background

The aim of preimplant surgery is the creation of an environment that is favorable to the function and long-term survival of endosseous dental implants. One essential requirement for successful implantation is the presence of sufficient bone in which the implants are placed. Besides the quantity of bone, the quality of bone and the intermaxillary relation play an important role [1]. Due to extremly atrophied alveolar process of the maxilla (class VI according to the classification of Cawood and Howell [2]) most patients suffer from a sagittal maxillary deficiency, a wide interarch distance and a reversed intermaxillary relationship giving patients an older appearance [3]. In these cases it is not sufficient to restore the lacking bone by onlay bone grafts or inlay bone grafts to the floor of the maxillary sinus [4], but to advise a simultaneous correction of the skelettal class-III conditions as described by Sailer 1989 [5].

The surgial approach of maxillary advancement is especially challenging in cleft patients due to the impared bony situation.

This report describes a methodological approach of treating a patient with severe mandibular and posterior maxillary alveolar atrophy and skelettal class-III conditions due to cleft palate performing sinus lifting and implant insertion before Le Fort I maxillary osteotomy.

## Case report

The patient was a 46-year-old man, afflicted with a cleft palate but no other serious diseases, when he first came to our departement for consultation complaining his loose fitting denture and asking for prosthetic treatment.

**Figure 1:**
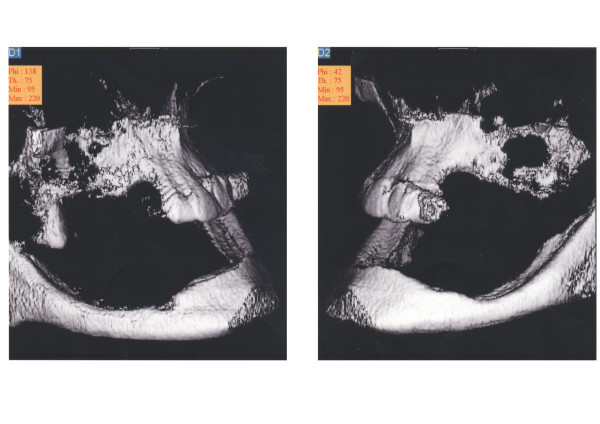
Preoperative 3D DVT-scan (digital volume tomography, New Tom 9000, New Tom Marburg, Germany), right view (left) and left view (right): initial bony situation: moderately severe atrophied mandible, severe posterior maxillary alveolar atrophy and skelettal class-III conditions due to cleft palate.

**Figure 2:**
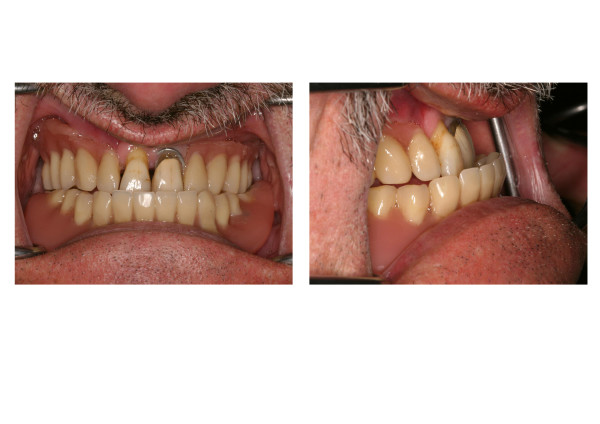
Preoperative clinical situation front view (left) and lateral view (right): noticeable class-III occlusion.

Clinical and radiographic examination (including 3D DVT scan [digital volume tomography, New Tom 9000, New Tom Marburg, Germany]) revealed an edentulous moderately severe atrophied mandible, a partialy edentulous maxilla, with severe posterior maxillary alveolar atrophy and skelettal class-III conditions due to cleft palate (figures 1 and 2).

In March 2004 extraction of the teeth 12, 17, 22, bilaterally sinus lifting procedure and a simultaneous alveolar ridge augmentation of the maxilla and the mandible were peformed under general anaesthesia. A mixture of cancellous bone from the iliac crest and Grafton^®^-DBM-Putty (Osteotech, Eatontown, NJ, USA) was used for the maxillary sinus floor augmentation. The lateral augmentation was performed using screw fixed autogenous corticocancellous block grafts and particulate bone grafts from the iliac crest mixed with Grafton^®^-DBM-Putty (Osteotech, Eatontown, NJ, USA). To fulfill the patient's desire the teeth 11 and 21 were left in the maxilla.

After three months screws were removed and auxiliary implants placed in the mandible.

6 weeks later screws were removed from the maxilla and using preoperative fabricated surgical guides a total of 12 endosseous Camlog^® ^implants were accurately positioned in the mandible and the maxilla according to the predefined planning that was made up of DVT scan and a wax up. Again bone augmentation around the dental implants was performed using filter collected bone and a bioresorbable collagen membrane (BioMend Extend^®^, Zimmer Dental, Carlsbad, CA, USA).

**Figure 3:**
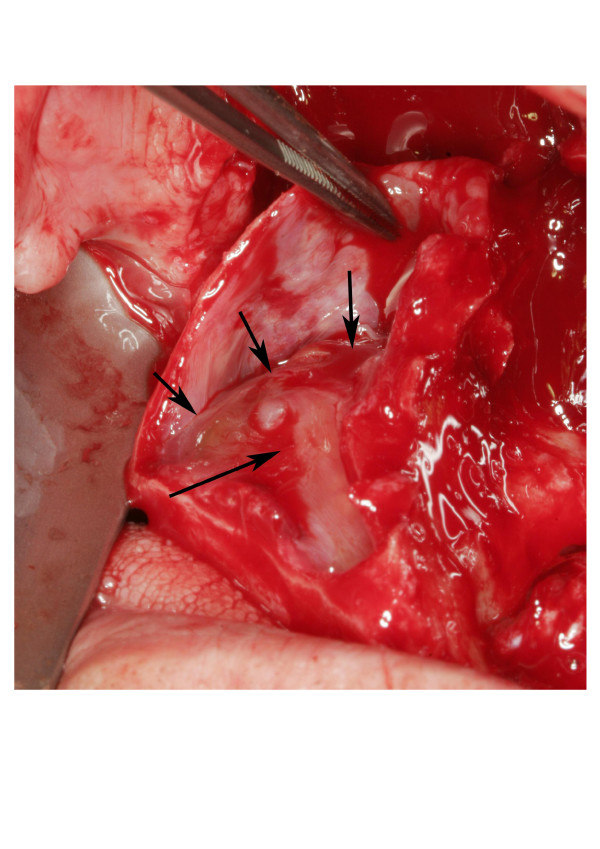
View from above to the grafted right maxillary sinus. Top of the former sinus augmentation (arrows)

**Figure 4:**
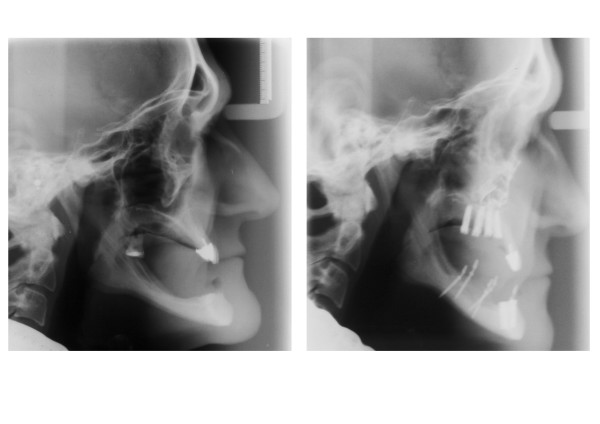
Preoperative (left) and postoperative (right) radiographs: improvement of the intermaxillary relationship after orthognathic surgery.

Based on the ideal implant position temporary protheses were fabricated and used for performing the modell-operation to correct the maxillo-mandibular relation. Three months later Le Fort I osteotomy with high horizontal bone cut was performed under general anaesthesia. The carefully downfractured maxilla allowed an unique view from above to the grafted sinuses (figure 3). The grafted bone showed high consistency and stability and except for a small mucocele in the right sinus no signs of any inflammatory irritation were detected. According to the preoperative planning the maxilla was placed in the new advanced position and then fixed with microplates (figure 4).

Postoperative healing was uneventful, except for a local infection that occurred two month later in the left upper canine region therefore the implant there had to be removed.

**Figure 5:**
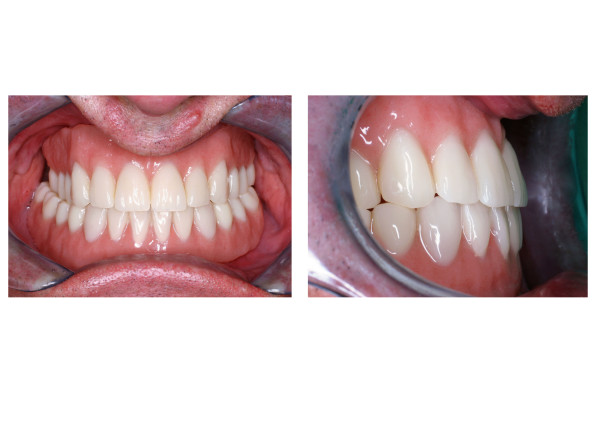
Postoperative clinical situation front view (left) and lateral view (right): noticeable class-I occlusion.

Six month later after extraction of teeth 11 and 21 the miniplates were removed from the maxilla and the implants uncovered. In addition abutments with protective healing caps were installed. After placing definite abutments and removing the auxililary implants final restauration was placed (figure 5).

## Discussion

The main basic criteria for restauration of the edentulous maxilla and mandible are adequate bone mass and ortholalveolar form [6]. This can be achieved by augmentation of the available substrate using established techniques such as vertical and lateral augmentation of the alveolar ridge, sinus floor bone grafting and orthognathic surgery [5,7-9]. Dependend on the initial situation one or more of these options can be used to improve load-bearing capacity for implants, whereas the use of vertical alveolar grafting for augmentation without implant placement is ineffective for bone mass maintenance in the long run [6,10].

Orthoalveolar form is the concept for optimal restauration of the edentulous alveolar ridge and means an idealized alveolar bone positioned in class I relation axially aligned to the opposing arch [6].

The resorptive pattern of the edentulous maxilla and mandible often leads to a discrepancy between the jaws such that a significant class III malocclusion occurs [11].

Edentulous patients with a skelettal class III jaw relationship have a poor chance of successful oral rehabilitation if they are provided exclusively with implant-supported prostheses unless supplementary surgery is also provided [5,12,13]. Implant-retained overdentures in fact offer the feasibility to compensate the retruded maxilla by placing prosthetic teeth anterior to the maxillary alveolar process, but that means a loss of the advantages of fixed tissue-integrated protheses, which have been described in longitudinal studies [13,14].

Sailer published a method of Le Fort I osteotomy in combination with simultanously bone grafting in the anterior and posterior maxilla and placement of endosseous implants for treatment of patients with atrophied maxillary alveolar bone and class III jaw relationship [5]. This sandwich technique permits simultaneous correction of the sagittal intermaxillary relationship and the vertical dimension. Some authors emphasize the advantages and satisfactory long-term results of the one stage procedure [4,15], but others prefer the two stage method as the long-term results are slightly superior to the one step procedure and simultanous insertion of endosseous implants increases the risk of bone necrosis and makes it difficult to achieve optimal position and angulation of the inserted implants [16-18].

A similar variant of the maxillary sandwich osteotomy for the rehabilitation of the severely atrophied maxilla is the horseshoe Le Fort I osteotomy where the horseshoe-shaped alveolar ridge is moved down and anterior after osteotomy and the hard palate remains pedicled on the nasal septum and vomer [3,19-24]. This technique is indicated in cases with flat palatal vault as the hard palate is not relocated and only the alveolar crest is moved in a favorable place thus resulting in a well shaped palatal vault that helps avoiding speech impairment and tongue displacement [3]. Analysis of the long term implant survival rate after one- or two stage implant insertion in the augmented maxilla showed no statistically significant differences [3,24].

Recently the concept of horizontal distraction osteogenesis for treatment of the atrophied anterior maxilla in combination with bilateral sinuslift operation was published [25]. The authors presented good results of implant osseointegration in the distracted bone during a follow up period of one year. They emphasize the alternative technique for correction of the interalveolar incongruences in the edentulous maxilla and augmentation prior to implant placement. However the main disadvantage of distraction osteogenesis is the need for enough bone as basis for regeneration and fixation of a stable distractor.

In our patient we found a moderately severely atrophied mandible and severely atrophied posterior maxilla and a skelettal class III jaw relationship amongst others due to the cleft palate. Minor degree maxillary alveolar atrophy was found in the anterior maxilla because of the still remaining teeth there.

The first step of our treatment concept was to reconstruct adequate bone mass by bilateral sinus lifting and onlay bone graft in the mandible and maxilla. On the way to configure ortholalveolar form we first placed the endosseous implants and than performed a classic Le Fort I osteotomy as described by Bell et al. [26]. Planning of orthognathic surgery was carried out on the basis of the implant borne temporary prostheses in ideal position.

The new method described is particularly recommendably to treat patients with atrophic maxilla and mandibula and a skelettal class III jaw relationship but minor degree vertical deficiency.

The advantages of our stepwise treatment are:

1. classic sinus lifting can be performed with a nearly predictible good result

2. two stage implant insertion offers better placemet opportunities and proper implant stability than the one stage procedure

3. implant placement before maxillary osteotomy avoids bone loss resulting from an extensive healing period and permits favorable conditions for exact adjustment of the postoperative prosthetic outcome

4. implants can be used for exact planning orthognatic surgery

5. classic le Fort I osteotomy can be performed, the previous sinus lifting stabilizes the fragile edentulous maxilla and reduces the risk to fracture

6. implants can be early loaded after healing period of orthognathic surgery is completed

The disadvantages of the treatment are:

It is a longsome treatment that requires at least two surgical procedures under general anaesthesia and the removal of bone from the iliac crest. Different from the method described by Sailer [5] our technique permits correction of the sagittal intermaxillary relationship but no gain of bone height in the vertical dimension.

## Conclusion

Sinus lifting before Le Fort I maxillary osteotomy is a particularly suitable method for oral rehabilitation of edentulous patients with skelettal class-III conditions and minor degree vertical deficiency especially in malformed patients.
